# Design and Implementation of a Novel System for Correcting Posture Through the Use of a Wearable Necklace Sensor

**DOI:** 10.2196/12293

**Published:** 2019-05-28

**Authors:** Hung-Yuan Chung, Yao-Liang Chung, Chih-Yen Liang

**Affiliations:** 1 Department of Electrical Engineering National Central University Taoyuan Taiwan; 2 Department of Communications, Navigation and Control Engineering National Taiwan Ocean University Keelung Taiwan

**Keywords:** wearable sensing technology, necklace, posture correction, image recognition, internet of things

## Abstract

**Background:**

To our knowledge, few studies have examined the use of wearable sensing devices to effectively integrate information communication technologies and apply them to health care issues (particularly those pertaining to posture correction).

**Objective:**

A novel system for posture correction involving the application of wearable sensing technology was developed in this study. The system was created with the aim of preventing the unconscious development of bad postures (as well as potential spinal diseases over the long term).

**Methods:**

The newly developed system consists of a combination of 3 subsystems, namely, a smart necklace, notebook computer, and smartphone. The notebook computer is enabled to use a depth camera to read the relevant data, to identify the skeletal structure and joint reference points of a user, and to compute calculations relating to those reference points, after which the computer then sends signals to the smart necklace to enable calibration of the smart necklace’s standard values (base values for posture assessment). The gravitational acceleration data of the user are collected and analyzed by a microprocessor unit-6050 sensor housed in the smart necklace when the smart necklace is worn, with those data being used by the smart necklace to determine the user’s body posture. When poor posture is detected by the smart necklace, the smart necklace sends the user’s smartphone a reminder to correct his or her posture; a mobile app that was also developed as part of the study allows the smart necklace to transmit such messages to the smartphone.

**Results:**

The system effectively enables a user to monitor and correct his or her own posture, which in turn will assist the user in preventing spine-related diseases and, consequently, in living a healthier life.

**Conclusions:**

The proposed system makes it possible for (1) the user to self-correct his or her posture without resorting to the use of heavy, thick, or uncomfortable corrective clothing; (2) the smart necklace’s standard values to be quickly calibrated via the use of posture imaging; and (3) the need for complex wiring to be eliminated through the effective application of the Internet of Things as well as by implementing wireless communication between the smart necklace, notebook computer, and smartphone.

## Introduction

### Background

Apple released the iPhone 3G, the world’s first 3G-capable phone, in 2008, thus ushering in the 3G revolution that has since significantly altered the lifestyles of phone users around the world, with countless users effectively becoming smartphone addicts. Unfortunately, such users may develop bone spurs in their cervical vertebrae because of the extended periods they spend with their backs in a slouched position while staring down at their phones. This, in turn, has meant that those patients seeking treatment for cervical degeneration have been of younger and younger average ages in recent years. Moreover, the postural problems associated with smartphone use can also negatively affect the pelvis and the caudal, thoracic, and lumbar vertebrae, with these negative effects potentially leading, in turn, to functional leg length discrepancies, scoliosis, and distended stomachs. Typically, posture correction efforts have utilized 1 of 2 approaches, with the approach used being determined according to the severity of the given patient's condition. Specifically, kyphosis correction belts (which consist of highly elastic fabrics and are used to provide support to the back) and kyphosis correction exercises may be utilized for patients with only mild cases of kyphosis, whereas rigid back braces made with aluminum alloys are more likely to be used to support and maintain back straightness in patients with severe spinal diseases.

Information communication technologies relating to newly developed wearable sensing devices have been touted as key technologies that can be applied in the health care field, and the use of these advanced technologies to address the aforementioned issues will become a trend going forward.

### Related Work

In recent years, the availability of increasingly smaller chips and greater computer power has accelerated the pace of development for wearable sensing devices. These developments have increased the applicability of such devices in the health monitoring and medical care field. Furthermore, wearable sensing devices also offer a considerably wide variety of application possibilities in other fields, such as force feedback devices, solutions for communication between people, environmental obstacle detection, and human-machine interface control.

In the health monitoring and medical care field, a study by Fallahzadeh et al [1] has examined the use of socks (embedded with accelerators and flexible stretch sensors) to detect ankle edema. In a study by Dauz et al [2], sleep quality was monitored by measuring skin potential activity, body temperature changes, and heart rates, and electrocutaneous stimulation was applied to the skin during the slow wave sleep stage to improve sleep quality. Sundaravadivel et al [3] and Boateng et al [4] explored the use of triple axis accelerometer readings to monitor the daily physical activity levels of individuals. In the study by Sundaravadivel et al [3], the data that were obtained were used to generate peak, mean, and standard deviation values, which were in turn used to differentiate between activities such as walking, stair climbing, and lying down. In the study by Boateng et al [4], a lightweight machine learning algorithm was used to analyze the obtained data and differentiate between the activities performed by users, so as to monitor and encourage physical activity among users. Liu et al [5] utilized accelerometers to measure electrocardiography signals and analyze user behavior. Martin and Voix [6] measured heart and respiratory rates by detecting sounds generated in the ear canal. Surrel et al [7] proposed a set of wearable devices capable of detecting sleep apnea. Takei et al [8] developed a wearable sensing device that can provide microelectric stimulation to the muscles and monitor muscle activity. Durbhaka [9] utilized shirts and pants embedded with triple axis accelerometers to analyze and assess human posture. Moreover, Gia et al [10] proposed a low-cost health monitoring system that involves the combined application of the Internet of Things with energy-saving sensor nodes and fog layers. This system utilized fog computing to automate services such as data sorting and channel management, allowing physicians to remotely monitor their patient’s physical conditions.

When wearable sensing devices are used as force feedback devices, they can enhance the virtual reality experience and convey the data collected by the sensors of robots as tactile feedback to users. For example, Chinello et al [11] designed a wearable fingertip cutaneous device that utilized 3 servo and vibration motors to create a tactile sensation at the fingertips of users. Through the use of motor-driven belts, Meli et al [12] were able to design a wearable sensing device for the upper limb that was capable of controlling the movements of a robotic arm and of receiving movement resistance feedback from the robotic arm. Wearable sensing devices can also be used to address communication problems between people. Goncharenko et al [13] analyzed images to identify sign language and enable the instant translation of the said language into a textual or auditory output, such that a user is able to communicate with a deaf-mute individual. Furthermore, wearable sensing devices can be used to detect environmental obstacles. Through the use of shoes equipped with ultrasonic sensors and vibration motors, Patil et al [14] were able to develop a navigational aid for individuals suffering from amblyopia and blindness. When a user’s foot comes too close to an obstacle, the shoes’ vibration motors will alert the user to the situation. With respect to the use of human-machine interfaces to change traditional mouse-based controls for computers, Zhang et al [15] examined the use of pressure sensors to detect the distribution of wrist strength and control cursor movements.

However, although all of these studies [1-15] did make significant contributions in various respects, none examined the use of wearable sensing devices to effectively integrate information communication technologies and apply them to health care issues (particularly those pertaining to posture correction).

### Study Objective

Motivated by the aforementioned lack of research, the objective of this paper was to design and implement a novel system for correcting posture that involved the use of a wearable necklace sensor and the integration of key emerging information communication technologies. The system was created with the aim of preventing the unconscious development of bad postures (as well as potential spinal diseases over the long term).

## Methods

### Overall Hardware Architecture

**Figure 1:**
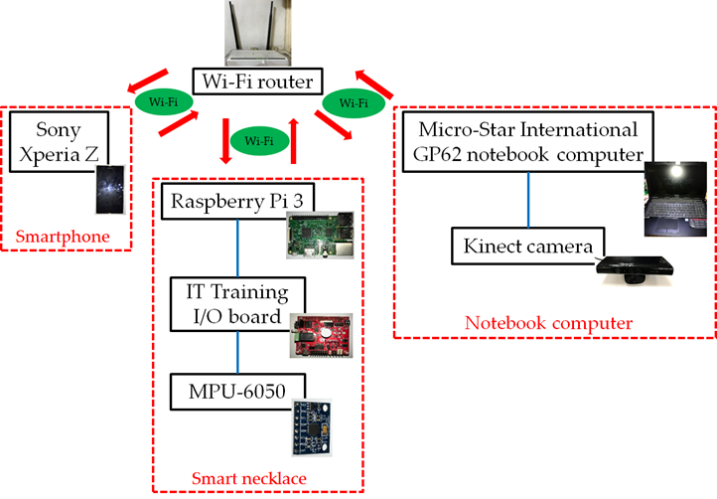
Hardware architecture of the overall system.

Figure 1 shows the overall hardware architecture proposed in this study. This architecture consists of 3 subsystems, namely, a smart necklace, notebook computer, and smartphone. The hardware specifications of the subsystems are described below:

1. Smart necklace: A Raspberry Pi 3 connected to an IT TRAINING [16] I/O board and, subsequently, to a Microprocessor Unit (MPU)-6050 sensor (the Raspberry Pi 3 requires a 5V power supply, which can be provided via a mobile power bank or transformer).
2. Notebook computer: A Micro-Star International GP62 notebook computer connected to a Kinect v1 camera [17].
3. Smartphone: A Sony Xperia Z with an Android operating system.

These 3 subsystems communicate through a Wi-Fi router. Data transfers between these 3 subsystems are carried out via the transmission control protocol/Internet Protocol (IP) network protocol. The mobile app developed in this study enables signal transmissions among the smartphone, the smart necklace, and the notebook computer. To facilitate later descriptions, the 2 primary transmission signals in this system are defined below:

1. Setting signals: These are signals (originating from the smartphone or notebook computer) used to adjust the smart necklace’s internal parameters.
2. Reminder signals: These are signals sent from the smart necklace to the smartphone, for the purpose of reminding the user to adjust his or her posture.

Furthermore, it should be noted that the activation of the overall system does not imply that the subsystems are now connected to each other. A connection between the transmitting and receiving ends is only established when the former intends to transmit a setting signal, and the connection is broken off when the transmission is completed.

### Overall System’s Operations

**Figure 2:**
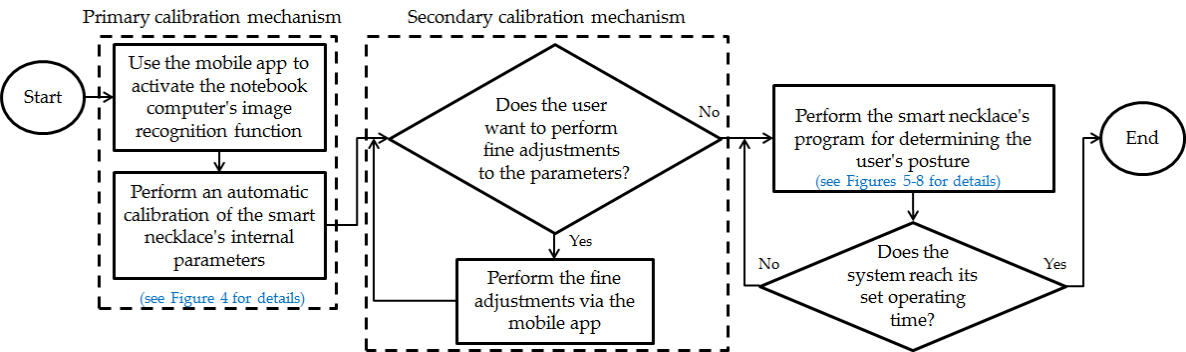
Outline flowchart of the overall system's operations.

Figure 2 is an outline flowchart that describes the overall system’s operations. After a new user puts on the smart necklace, its internal parameters have to be recalibrated. The overall system is first activated, after which the mobile app is used to activate the notebook computer’s image recognition function, which will then perform an automatic calibration of the smart necklace’s internal parameters (primary calibration mechanism). Following this, the user may opt to perform fine manual adjustments to the smart necklace’s internal parameters (secondary calibration mechanism), in which case he or she may do so via the smartphone. If the user opts not to make the above adjustments, the smart necklace’s program for determining the user’s posture will start. Subsequently, the mobile app is used to activate the function of receiving reminder signals from the smart necklace. The smart necklace will simultaneously be continuing its assessment of the user’s posture. In the event that poor posture is detected, a reminder signal will be sent to the smartphone. Once the system reaches its set operating time, its operations will end (the overall system shuts down).

**Figure 3:**
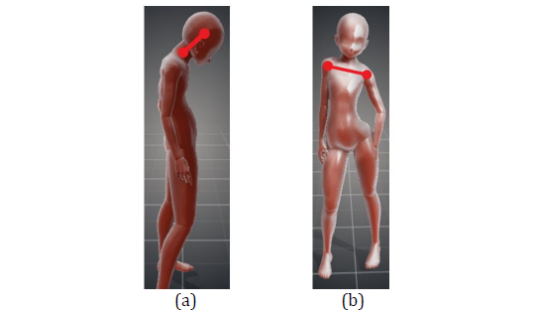
(a) Kyphosis and (b) left shoulder tilted downward.

**Figure 4:**
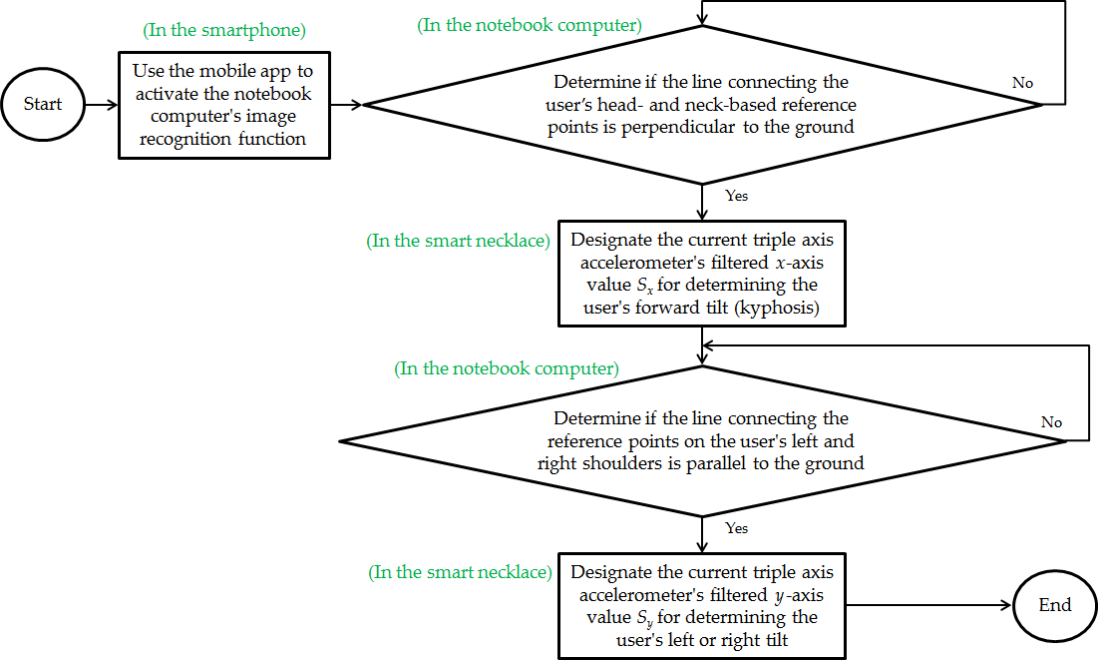
Interrelationship flowchart of the 3 subsystems when a new user utilizes the notebook computer’s image recognition function to perform an automatic calibration of the smart necklace’s internal parameters.

When diagnosing spine-related diseases, an orthopedist first performs a visual diagnosis. To determine if a patient is suffering from kyphosis, the orthopedist will use the center points of the patient’s head and neck as reference points and verify if the line connecting these 2 points is perpendicular to the ground (a kyphotic posture in Figure 3). As for the diagnosis of scoliosis, the orthopedist will use the left and right shoulder joints as reference points and verify if they are at an even height (a posture in which left shoulder is tilted downward in Figure 3). Thus, these criteria were used in the study as the basis for determining if the body posture of a person is poor. To facilitate later descriptions, we defined the variable *S*_*i*_ (unit: G) as the gravitational acceleration value (a reference value for determining posture) for the direction *i*, with *i*= *x* representing the front-back direction and *i*= *y* representing the left-right direction, measured by the MPU-6050 when a user has a standard posture. *S*_*i*_ will change according to the notebook computer’s image recognition results (see Figure 4) or the fine manual adjustments to the parameters made via the smartphone (see the Smartphone section).

Figure 4 shows an interrelationship flowchart of the 3 subsystems when a new user utilizes the notebook computer’s image recognition function to perform an automatic calibration of the smart necklace’s internal parameters. The user first activates the smartphone’s automatic calibration function, which then activates the notebook computer’s skeletal structure image recognition function. Following this, the notebook computer assesses the user’s side posture by determining if the line connecting his or her head- and neck-based reference points is perpendicular to the ground. When the line is not perpendicular to the ground, the notebook computer will continue with the image assessment process. When the line is perpendicular (indicating proper posture), the notebook computer will send a signal to the smart necklace, where the triple axis accelerometer’s *x*-axis value will be digitally filtered (the current value and the previous values are averaged) and designated as the standard value *S*_*x*_ for determining the user’s forward tilt (kyphosis). Next, the notebook computer will assess the user’s frontal posture to determine if the line connecting the reference points on the user’s left and right shoulders is parallel to the ground. If the line is not parallel to the ground, the image assessment process will continue. Conversely, if the line is parallel to the ground, the notebook computer will send a signal to the smart necklace, where the triple axis accelerometer’s *y*-axis value will be digitally filtered and designated as the standard value *S*_*y*_ for determining the user’s left or right tilt. After the internal parameters of the smart necklace have been successfully calibrated, the smartphone will receive a message informing the user of the successful calibration. At this point, the program running on the notebook computer will close.

In the following sections, the design of the smart necklace, operation of the notebook computer, and design and operation of the smartphone will be described.

### Smart Necklace

**Figure 5:**
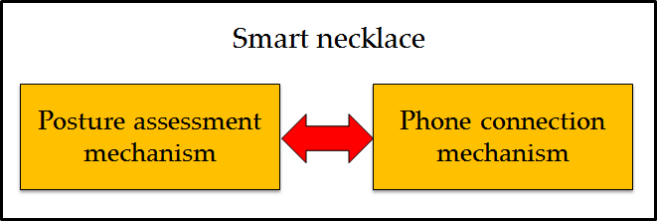
Two mechanisms of the smart necklace, namely, the posture assessment and phone connection mechanisms, responsible for performing posture assessments and sending reminder signals to the smart phone, respectively.

Overall, 2 mechanisms were incorporated into the design of the smart necklace, namely, the posture assessment and phone connection mechanisms, which operate concurrently (see Figure 5). The posture assessment mechanism allows for the posture analysis of the gravitational acceleration data recorded by the MPU-6050, whereas the phone connection mechanism allows for reminder signals to be sent to the smartphone.

#### Posture Assessment Mechanism

**Figure 6:**
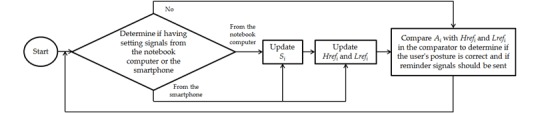
Flowchart describing the posture assessment performed by the smart necklace.

**Figure 7:**
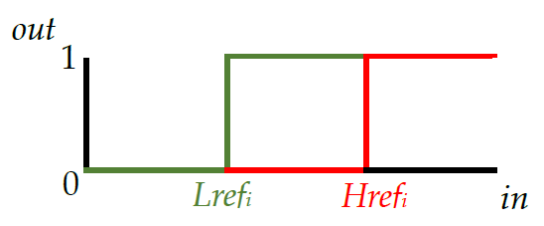
Hysteresis comparator.

Figure 6 is a flowchart describing the posture assessment performed by the smart necklace. The core of the assessment involves the use of a digital hysteresis comparator (see Figure 7), which contains 4 key elements, namely, the input (*in*), output (*out*), and 2 thresholds (*Lref*_*i*_ and *Href*_*i*_). It should be noted that Figure 6 implicitly shows 3 comparators, which are used for the frontal, left, and right tilt values. Here, the left and right tilt indexes pertaining to the thresholds are expressed as *yl* and *yr*, respectively. We defined *A*_*i*_ as the value obtained after digitally filtering the triple axis accelerometer’s continuously changing *i*-axis value (note that *S*_*i*_ is the *A*_*i*_ when the user's posture is correct). The Raspberry Pi 3 utilizes an I2C transfer protocol to read the data and compare the digitally filtered values (ie, *A*_*i*_) with the thresholds (*Lref*_*i*_ and *Href*_*i*_), so as to determine if the user’s posture is correct. In other words, *A*_*i*_ is substituted into *in*. When *out* is equal to 0, no reminder signals will be sent. However, when *in* becomes higher than *Href*_*i*_, the value of *out* then becomes 1 and a reminder signal (indicating that the smart necklace has detected poor posture) will be sent via the phone connection mechanism. When *out* is initially equal to 1, a reminder signal will be sent. However, when in is lower than *Lref*_*i*_, the value of *out* then becomes 0 and no further reminder signals will be sent (indicating that the smart necklace has determined the user’s posture to be correct).

Each individual’s skeletal structure is different, and shoulder width is an important indicator that helps to define the characteristics of a skeletal structure. On the basis of the shoulder width characteristics of a normal individual, 3 users (whose shoulder widths were generally considered to be short, moderate, and long) were selected for the tests. To some degree, this selection method allowed us to cover the entire range of normal skeletal structures. Thus, this design approach should be valid for the general population (however, the system’s threshold values must be set separately for skeletal structures with abnormal characteristics, and it is recommended that future studies could focus on this aspect of the research).

The threshold values were set based on posture assessments of the 3 users (indexed by *j*, with *j*=1, 2, 3) that were performed by 3 orthopedists. First, a user would tilt forward until the orthopedist determines that a poor posture has been achieved, at which point the triple axis accelerometer’s digitally filtered *x*-axis value (ie, the value 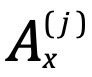) would be recorded. Next, the user would tilt leftward until the orthopedist determines that a poor posture has been achieved, at which point the triple axis accelerometer’s digitally filtered *y*-axis value (ie, the value 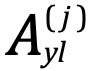) would be recorded. Finally, the user would tilt rightward until the orthopedist determines that a poor posture has been achieved, at which point the triple axis accelerometer’s digitally filtered *y*-axis value (ie, the value 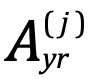) would be recorded. On the basis of the standards set by the 3 orthopedists, the values (ie, 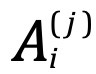, *i=x, yl, yr*) recorded for the 3 users are presented in Tables 1-3, respectively, with | 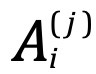
− 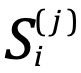
| representing the change in user *j*’s tilt toward direction *i* at the point when the value was recorded.

To implement stricter posture assessment standards for the system, the value (min_*j*__=1, 2, 3_ | 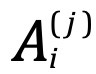
− 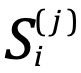
| ), which represents the minimal degree of change, was used as the basis for the assessment. In other words, even a slight tendency to tilt will be identified as poor posture by the smart necklace, which will send a reminder to the user to prompt a correction.

More specifically, Tables 1-3 show that the minimum value of the change in the 3 users’ forward tilt was 0.01159G. Thus, *Href*_x_ was defined as:

*Href_x_*=*S_x_*–0.01159 [1]

To prevent the smart necklace from sending reminders at an erratic frequency, numerous rounds of data evaluation were performed, and the value 0.00854G (which is approximately equal to the value of 0.01159G minus 0.003G) was selected for use to set *Lref*_x_. Thus, *Lref*_x_ was defined as:

*Lref_x_*=*S_x_*–0.00854 [2]

**Table 1:** Measurement results of user number 1 based on orthopedists’ standards.

Model	Value (G)	Change (G) | 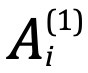 − 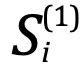 |
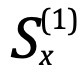	0.99288	—^a^
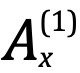	0.93354	0.05934
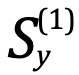	−0.06689	—
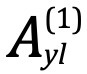	0.00896	0.07585
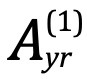	−0.17777	0.11087

^a^Not applicable.

**Table 2:** Measurement results of user number 2 based on orthopedists’ standards.

Model	Value (G)	Change (G) | 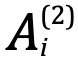 − 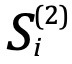 |
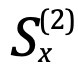	0.98999	—^a^
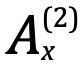	0.94897	0.04102
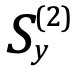	−0.16921	—
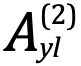	−0.14087	0.02834
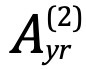	−0.21952	0.05031

^a^Not applicable.

**Table 3:** Measurement results of user number 3 based on orthopedists’ standards.

Model	Value (G)	Change (G) | 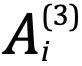 − 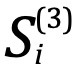 |
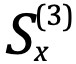	0.99846	—^a^
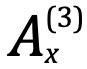	0.98687	0.01159
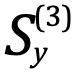	−0.06850	—
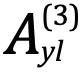	−0.01312	0.05538
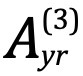	−0.12673	0.05823

^a^Not applicable.

Furthermore, the minimum value of the change in the 3 users’ left/right tilt was 0.02834G, hence the use of this value to set *Href*_*yl*_ and *Href*_*yr*_ and the selection of the value 0.02441 (which is approximately equal to the value of 0.02834G minus 0.004G) to set *Lref*_*yl*_ and *Lref*_*yr*_. Thus, *Href*_*yl*_, *Lref*_*yl*_, *Href*_*yr*_, and *Lref*_*yr*_ were set as:

*Href_yl_*=*S_y_*+0.02834 [3]

*Lref_yl_*=*S_y_*+0.02441 [4]

*Href_yr_*=*S_y_*–0.02834 [5]

and

*Lref_yr_*=*S_y_*–0.02441 [6]

#### Phone Connection Mechanism

**Figure 8:**
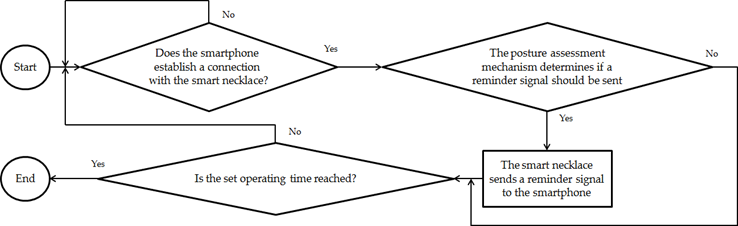
Flowchart describing the process whereby a reminder signal is sent from the smart necklace to the smartphone.

Figure 8 shows the process whereby a reminder signal is sent from the smart necklace to the smartphone. The smart necklace first waits for the smartphone to establish a connection with it. If a connection is not established, the smart necklace will continue to wait. If a connection is established, the posture assessment mechanism will be used to determine if a reminder signal should be sent (ie, the outcome described in the *Posture Assessment Mechanism* section). If the outcome involves the sending of a reminder, the smart necklace will send a reminder signal to the smartphone. The aforementioned process is repeated continuously to enable the continuous assessment of the user’s posture, that is, until the set operating time is reached and the system’s operation ends (the overall system shuts down).

### Notebook Computer

**Figure 9:**
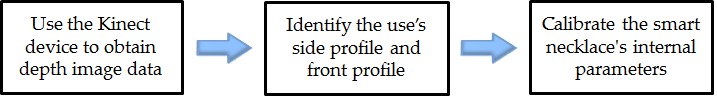
Operating process whereby the notebook computer is used to perform posture recognition.

Figure 9 shows the operating process whereby the notebook computer is used to perform posture recognition. Among the considerable number of methods used to detect human skeletal structures, the method proposed by Microsoft [18] was selected for this study. This method involves the use of the Kinect device to obtain depth image data, which are then analyzed using random decision trees and forests [19] to identify joint points. Once a user’s joint points are identified, he or she will turn to allow the camera to capture his or her side profile. Calculations are then performed to determine if the line connecting his or her head- and neck-based reference points is perpendicular to the ground, so as to determine the presence or absence of kyphosis. Next, the user will turn again to allow the camera to capture his or her front profile. Calculations are then performed to determine if the line connecting the user’s left and right shoulders is parallel to the ground, so as to determine if the user’s standing posture is correct. The use of this method makes it possible to perform posture assessments and, thus, calibrate the smart necklace’s internal parameters.

### Smartphone

**Figure 10:**
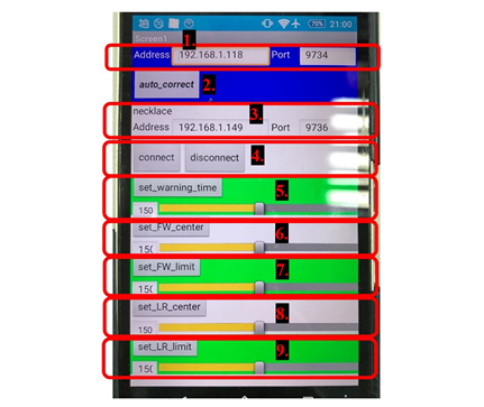
Mobile app interface.

The App Inventor 2 [20], which was developed by the Massachusetts Institute of Technology Center for Mobile Learning, is used in this study as it does away with the need to use Java to open up Blocks Editor (which is integrated into the website and can be used immediately) and makes it easy for developers to write Android apps. For the human-machine interface, 2 IPv4 addresses are required for signal transmission, with one serving as the IP address for the camera linked to the notebook computer and the other serving as the IP address for the smart necklace (see items 1 and 3 of Figure 10). The *auto_correct* button (ie, item 2 of Figure 10) is used to “activate the notebook computer’s image recognition function and the automatic calibration of the smart necklace’s internal parameters.” The *connect* and *disconnect* buttons (item 4 of Figure 10) are used to “enable the smartphone to connect to the smart necklace and receive reminder signals from the smart necklace” and “disconnect the smartphone and smart necklace (no reminder signals will be received),” respectively. Furthermore, it is possible to manually set the smartphone’s internal parameters using items 5 to 9 of Figure 10. The *set_warning_time* button (ie, item 5) is used to “adjust the frequency at which the smart necklace sends reminder signals when poor posture is detected (the higher the value, the higher the frequency).” The *set_FW_center* button (ie, item 6) is used to “set parameter *a* (adjustable range of 1-300 and central value of 150) so as to make minor adjustments to the *S*_*x*_ value of the smart necklace,” with the adjustment formula being:

*S_x_*=*S_x_*×(a/150) [7]

The *set_FW_limit* button (ie, item 7) is used to “set parameter *b* (adjustable range of 1-300 and central value of 150) so as to make minor adjustments to the *Href*_*x*_ and *Lref*_*x*_ values of the smart necklace,” with the adjustment formulas being:

*Href_x_*=*S_x_*–(0.01159×[b/150]) [8]

and

*Lref_x_*=*S_x_*–(0.00854×[b/150]) [9]

The *set_LR_center* button (ie, item 8) is used to “set parameter *c* (adjustable range of 1-300 and central value of 150) so as to make minor adjustments to the *S*_*y*_ value of the smart necklace,” with the adjustment formula being:

*S_y_*=*S_y_*×(c/150) [10]

The *set_LR_limit* button (ie, item 9) is used to “set parameter *d* (adjustable range of 1-300 and central value of 150) so as to make minor adjustments to the *Href*_*yl*_, *Lref*_*yl*_, *Href*_*yr*_, and *Lref*_*yr*_ values of the smart necklace,” with the adjustment formulas being:

*Href_yl_*=*S_y_*+(0.02834×[d/150]) [11]

*Lref_yl_*=*S_y_*+(0.02441×[d/150]) [12]

*Href_yr_*=*S_y_*–(0.02834×[d/150]) [13]

and

*Lref_yr_*=*S_y_*–(0.02441×[d/150]) [14]

It should be noted that the 4 parameters *a*, *b*, *c*, and *d* are independent of each other when the adjustments are being made.

Recall that formulas (1) to (6) involve the utilization of the notebook computer’s image recognition function to perform an automatic calibration of the smart necklace’s *S*_*i*_ value (primary calibration mechanism), whereas formulas (7) to (14) correspond to the fine manual adjustments to the smart necklace’s internal parameters that a user may want to further perform (secondary calibration mechanism). Thus, when formulas (7) to (14) are used, the *S*_*i*_, *Href*_*i*_, and *Lref*_*i*_ values that are already present in formulas (1) to (6) will be updated by formulas (7) to (14).

## Results

**Figure 11:**
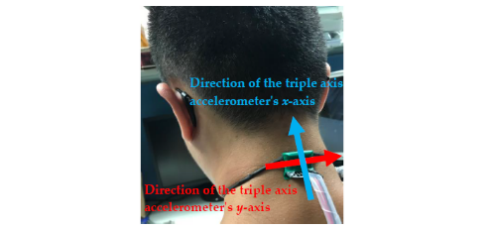
The position and appearance of the smart necklace when worn around the back of the user's neck, with the blue and red arrows indicating the directions of the triple axis accelerometer's *x*-axis and *y*-axis, respectively.

This section describes the practical tests performed on the proposed posture correction system (see Figure 1 for hardware specifications) to demonstrate its effectiveness. During the tests, the smart necklace was worn around the back of the user’s neck. The position and appearance of the smart necklace are shown in Figure 11. In addition, a demo video has been provided to further demonstrate the advantages of our system (see Multimedia Appendix 1).

**Figure 12:**
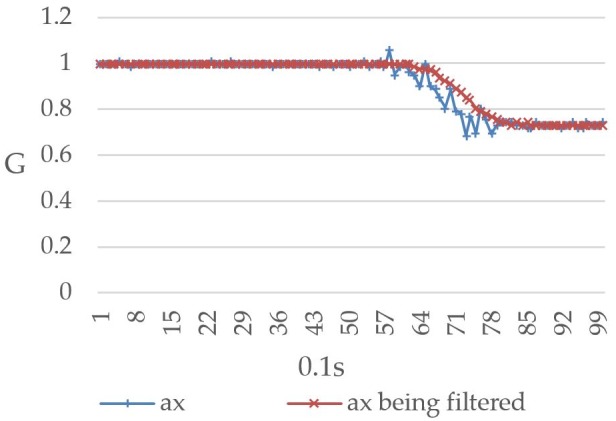
Triple axis accelerometer's pre-filtered *x*-axis values (ax) and filtered *x*-axis values (ax being filtered) when the user's body was tilted forward.

**Figure 13:**
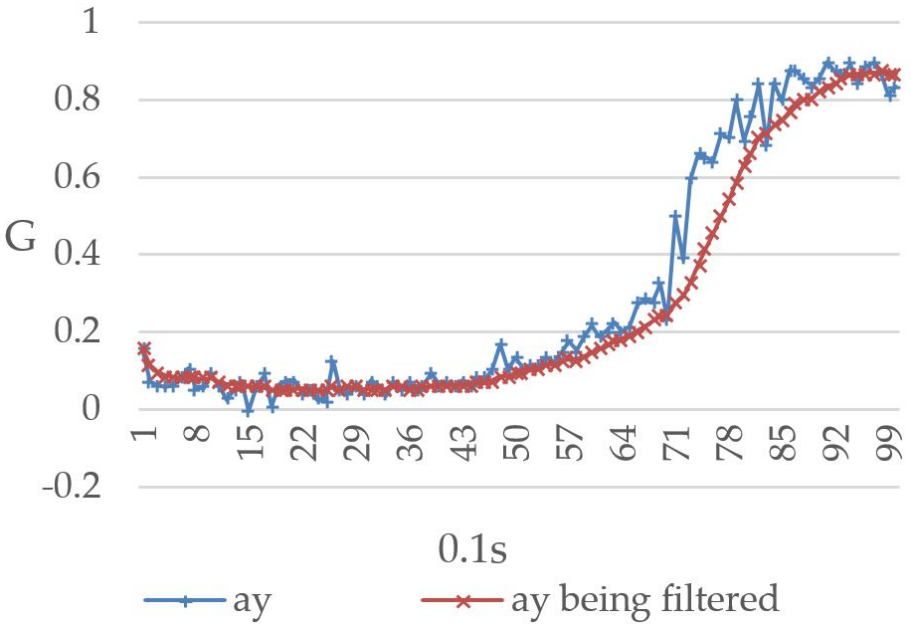
Triple axis accelerometer's pre-filtered *y*-axis values (ay) and filtered *y*-axis values (ay being filtered) when the user's body was tilted leftward.

**Figure 14:**
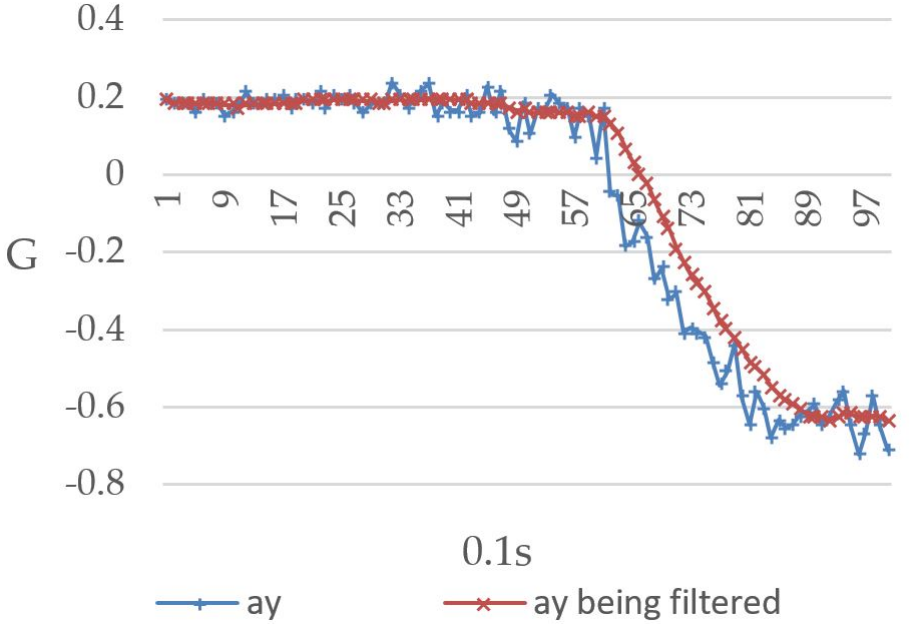
Triple axis accelerometer's pre-filtered *y*-axis values (ay) and filtered *y*-axis values (ay being filtered) when the user's body was tilted rightward.

**Figure 15:**
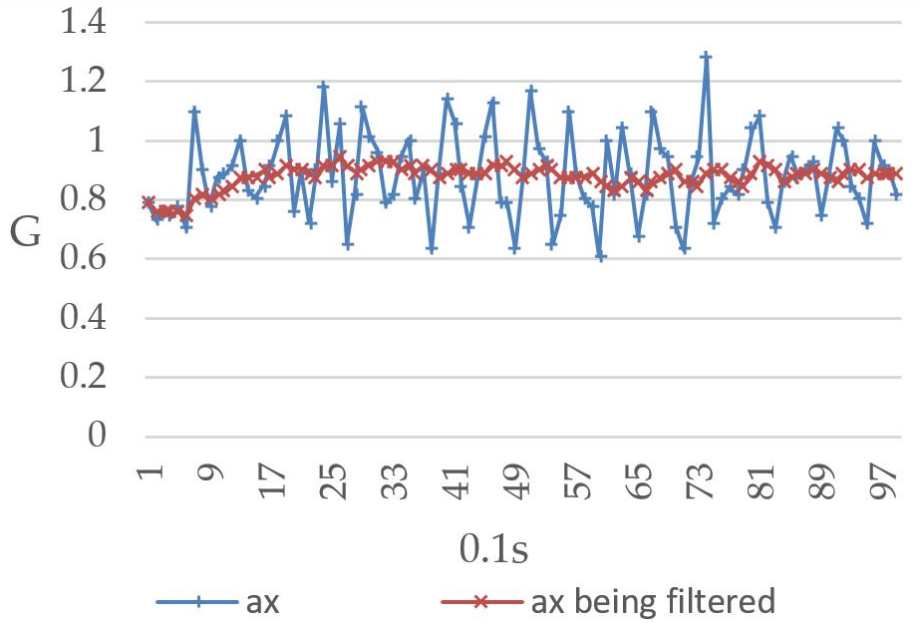
Triple axis accelerometer's pre-filtered *x*-axis values (ax) and filtered *x*-axis values (ax being filtered) under walking conditions.

The Raspberry Pi 3 reads the MPU-6050 value once every 0.1 seconds and repeats this process 99 times to record the relationship between the user’s body movement and the readings. Figure 12 shows the values recorded when the user’s body was tilted forward, including both the prefiltered and filtered values. The horizontal and vertical axes represent time (measured at 0.1-second intervals) and gravitational acceleration G, respectively, and ax represents the triple axis accelerometer’s *x*-axis value, which decreased from 1G to approximately 0.73G. Figure 13 shows the values recorded when the user’s body was tilted leftward, with ay representing the triple axis accelerometer’s *y*-axis value, which is shown to have increased from 0.1G to approximately 0.9G. When the user’s body was tilted rightward, the values changed in the opposite direction. As shown in Figure 14, the ay value decreased from 0.2G to around −0.6G. Furthermore, ax was affected to a greater degree than ay under walking conditions, which led to a considerable level of noise interference. To address this issue, both the prefiltered and filtered ax values were recorded to demonstrate the changes that occurred (see Figure 15).

Given that the values will become somewhat unstable when there is noise interference, the mean value of the current reading and the previous 9 readings was thus used in this study for the purpose of eliminating noise interference and preventing the system from making errors. Looking at Figures 12-15, it is clear that the filtering has caused the values to become more stable and reduced signal fluctuations.

### Automatic Calibration of Smart Necklace’s Internal Parameters Through the Notebook Computer’s Image Recognition Function

#### Measurements Performed on a Single User

**Figure 16:**
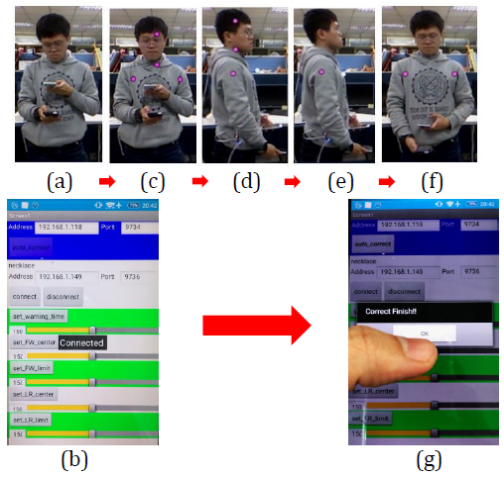
Program that utilizes the notebook computer's image recognition function to perform an automatic calibration of the smart necklace's internal parameters.

The user first activates the notebook computer (which is linked to a camera). Next, the mobile app is activated and the *auto_correct* button (see Figure 16) is clicked to activate the notebook computer’s image recognition function and perform the automatic calibration of the smart necklace’s internal parameters, after which the *Connected* status will be displayed on the smartphone. At this point, the notebook computer will attempt to detect the user’s skeletal structure and mark out the head, neck, left shoulder, and right shoulder positions using 4 pink points. The user first turns to allow the camera to capture his side profile, and when the line connecting his head- and neck-based reference points is perpendicular to the ground, the computer will transmit a setting signal to the smart necklace to calibrate *S*_*x*_ and set it as the standard value for determining the presence of kyphosis. Upon the calibration’s completion, the head- and neck-based points will disappear. Following this, the user then turns to face the camera frontally, and when the line connecting the reference points on the user’s left and right shoulders is parallel to the ground, the computer will transmit a setting signal to the smart necklace to calibrate *S*_*y*_ and set it as the standard value for determining the presence of a left/right tilt. Upon the calibration’s completion, the image program closes automatically and the *Correct Finish!!* status is displayed on the smartphone to indicate that the calibration has been completed.

#### Measurement Data of and Individual Differences Between 3 Users

Define 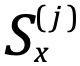
and 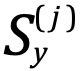, respectively, as the forward tilt and left/right tilt standard values of the 3 users (indexed by *j*, with *j*=1, 2, 3) who were tested. These are the values obtained after the notebook computer’s image recognition function was utilized to perform an automatic calibration of the smart necklace’s internal parameters as shown in Figure 2 (to facilitate the description of the core concepts, the mobile app was not used to perform fine manual adjustments in this scenario).

**Figure 17:**
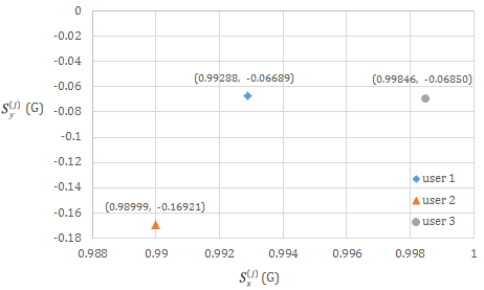
Distribution of the standard values of the 3 users (who were tested) across a 2-dimensional scatter plot.

Figure 17 is a 2-dimensional scatter plot showing the respective 
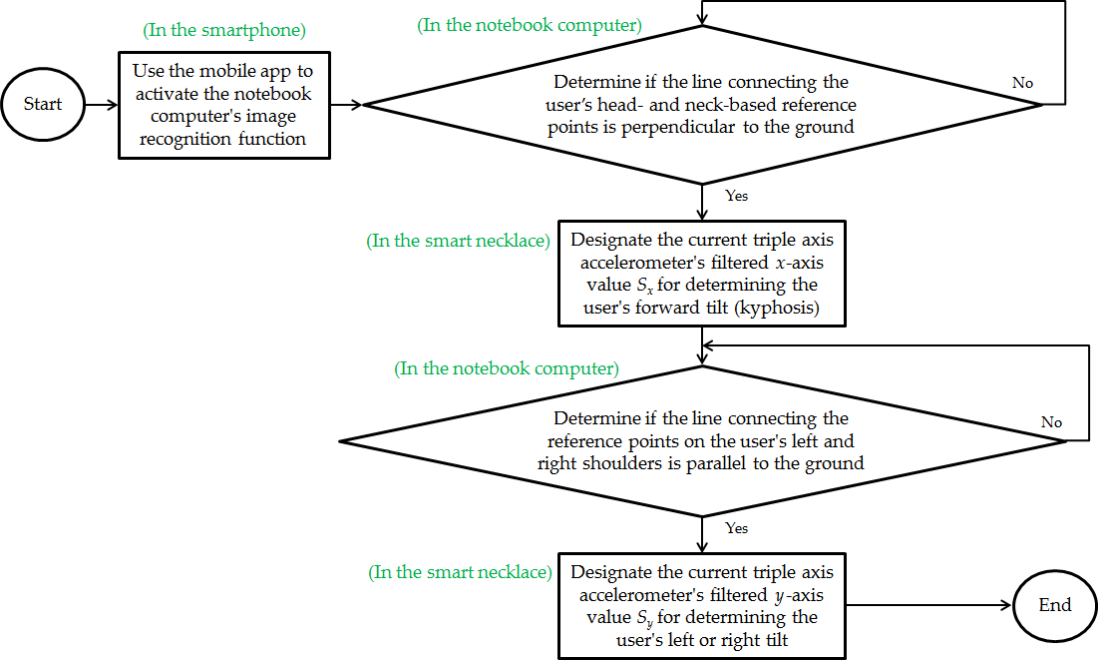
and 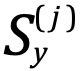
values of the 3 users. As shown in the figure, the coordinate points (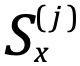, 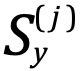) for each user’s body are different, primarily because skeletal structures differ from individual to individual.

Thus, a user new to the system would have to recalibrate his or her 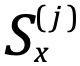
and 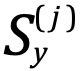
values to ensure that accuracy of his or her posture assessment.

### Manipulation of the Smart Necklace’s Internal Parameters via the Smartphone

#### Adjustment of the Smart Necklace’s Internal Parameters

**Figure 18:**
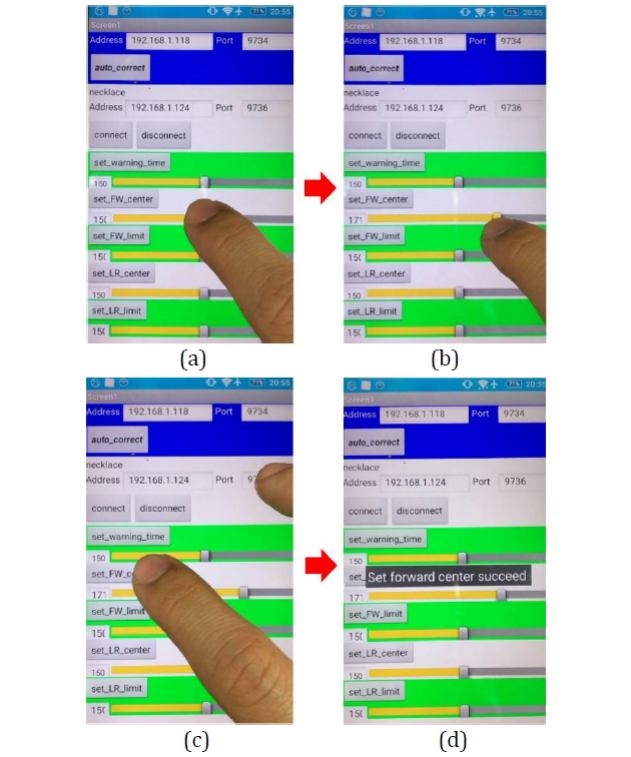
Setting of the smart necklace's internal parameters manually.

**Figure 19:**
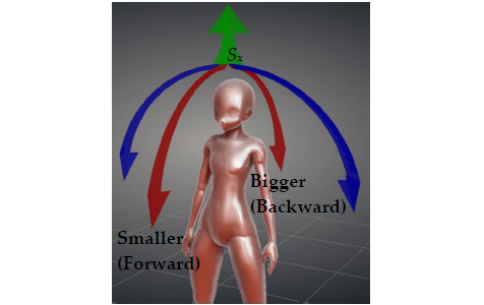
Diagram showing the correction of posture standard values.

This section describes the manner in which a user can make further fine adjustments to the smart necklace’s internal parameters. For example, the *S*_*x*_ value may be adjusted by dragging the corresponding horizontal slider to the desired value (see Figure 18) based on formula (7). The bigger this value is, the greater the extent to which the *S*_*x*_ value is being corrected toward the back (see the red arc in Figure 19 that is pointing toward “Backward”). Conversely, the smaller this value is, the greater the extent to which the *S*_*x*_ value is being corrected toward the front (see the red arc in Figure 19 that is pointing toward “Forward”). Subsequently, the *set_FW_center* button is clicked to transmit a setting signal to the smart necklace. This results in the appearance of the *Set forward center succeed* message on the screen of the smartphone, which indicates that the adjustment of *S*_*x*_ is complete.

#### Activation to Receive the Smart Necklace’s Reminders

**Figure 20:**
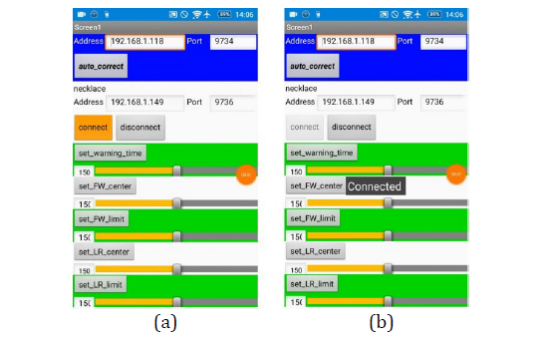
Activation of the smartphone’s function for receiving reminders from the smart necklace.

The mobile app is activated, and the *connect* button is clicked (see Figure 20) to link the smartphone and smart necklace. At this point, the *Connect* *ed* message will be displayed on the screen of the smartphone, which has now activated its function for receiving reminders from the smart necklace.

**Figure 21:**
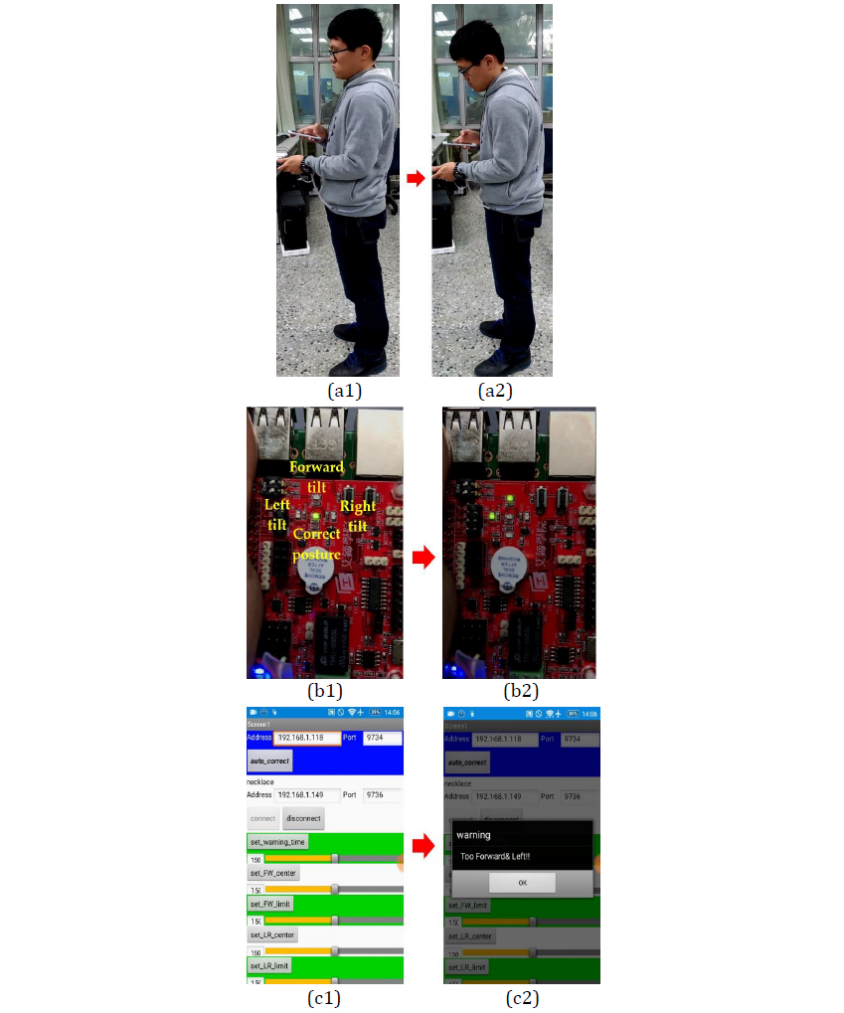
Posture with forward-left tilt.

When a user displays the tendency to shift from a correct posture to an incorrect one, the corresponding yellow Light-Emitting Diode (LED) on the IT TRAINING expansion board will light up. As shown in Figure 21, the IT TRAINING expansion board has 4 LEDs. The activation of the middle LED indicates correct posture, the activation of the top LED indicates a forward tilt, the activation of the left LED indicates a left tilt, and the activation of the right LED indicates a right tilt. For instance, when a user shifts from a correct posture to one with a left and forward tilt, the left and top LEDs on the IT TRAINING expansion board will light up simultaneously, whereas the middle LED will turn off. At the same time, the smart necklace will send a reminder signal to the smartphone, causing the *Too Forward & Left!!* reminder to appear on the screen of the smartphone. This serves to remind the user that he is displaying a forward-left tilt that requires correction.

**Figure 22:**
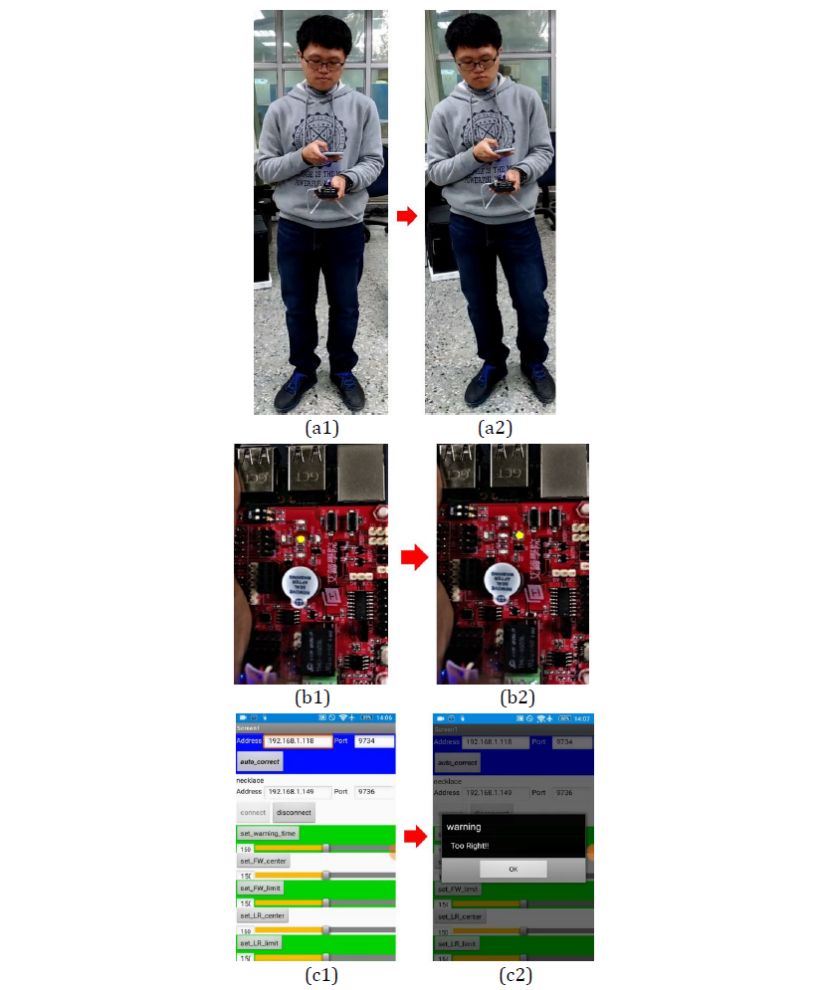
Posture with right tilt.

For the sake of completeness, another example is provided (see Figure 22). In this case, the user shifts from a correct posture to one with a right tilt; this change will be detected by the smart necklace and reflected on the IT TRAINING expansion board’s LEDs. In other words, the middle LED will turn off while the right LED will light up. At the same time, the smart necklace will send a reminder signal to the smartphone, causing the *Too Right!!* reminder to appear on the screen of the smartphone. This serves to remind the user that he is displaying a right tilt that requires correction.

**Figure 23:**
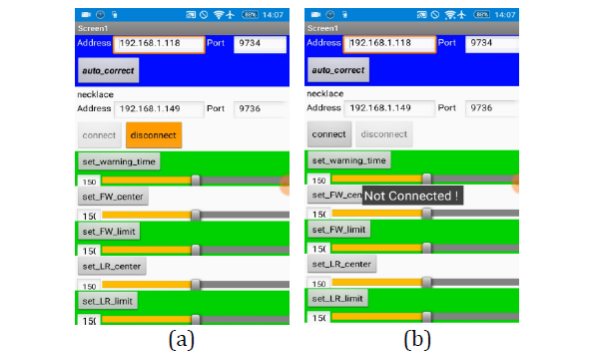
Deactivation of the smartphone’s function for receiving reminders from the smart necklace.

If a user does not want to receive any reminders regarding poor posture, he or she may click the *disconnect* button (see Figure 23) to cut off the connection between the smartphone and the smart necklace and cause the *Not Connect* *ed!* message to be displayed on the smartphone’s screen. At this point, the smartphone has already deactivated its function for receiving reminders from the smart necklace.

## Discussion

### Principal Findings

In this study, a novel posture correction system was developed through the application and integration of image recognition, wearable sensing, and Internet of Things technologies, with this new system consisting of 3 subsystems: a smart necklace, notebook computer, and smartphone. The system constitutes a pioneering achievement with respect to research on the application of wearable sensing devices in posture correction.

The notebook computer is equipped with a Kinect camera and can be used to calibrate the smart necklace and ensure that it does not lose its effectiveness in the event that it is improperly used. The posture image recognition function of the notebook computer gives the user the ability to quickly calibrate the internal parameters of the smart necklace subsystem. By wearing the lightweight smart necklace and carrying the smartphone with the mobile reminder system, the user is able to self-correct his or her posture and prevent spine-related diseases without the use of corrective clothing that is heavy, thick, and uncomfortable. Moreover, because the system utilizes wireless communication among the interfaces of its different subsystems, the user does not have to deal with complex wiring, thus allowing him or her to utilize the new system in a convenient manner.

### Limitations

The posture correction system’s 3 subsystems communicate with each other via a Wi-Fi router, making it suitable for office or home use. However, this design also requires the 3 subsystems to be located in close proximity to each other, so as to enable the near-instant transmission of signals between them. Using the system used in this study as a foundation, future studies could explore ways to enable users to perform instant posture correction regardless of their location (ie, via a system that can be used in any environment).

### Comparison With Prior Work

The developmental trends in information and communications technology are pointing toward the substantial application of technologies such as wearable sensors, image recognition, and the Internet of Things in smart medical systems. However, to our knowledge, few studies have looked into the effective integration and application of these technologies for posture correction. In addition, physicians typically do not recommend that patients suffering from only mild or temporary kyphosis symptoms make use of heavy, thick, or uncomfortable corrective clothing. At the same time, it is often difficult for patients who attempt to rely only on their own willpower to achieve good results. The system introduced herein was thus developed with these issues in mind to assist users in detecting poor posture, correcting their postures as appropriate, and, in turn, protecting themselves from spinal diseases resulting from the long-term effects of bad posture.

### Conclusions

The spine is the most important group of bones in the human body, as it supports not only the weight of the body but also enables it to perform the full range of torsional motions. When poor posture is maintained on a frequent basis or over a long period, issues including spine-related problems or even the onset of premature aging and geriatric diseases among young adults may occur. Thus, the most basic approach to protecting our spines consists of fixing our bad postures. With that in mind, the proposed system can effectively enable a user to monitor and correct his or her own posture, which in turn will assist the user in preventing spine-related diseases and, consequently, in living a healthier life. We believe that this system can contribute to our health and serve as a pioneering system with respect to the application of integrated forward-looking technologies to posture correction. Furthermore, this system is expected to meet current and future needs and possess commercial potential.

## Appendix

Multimedia Appendix 1 Demo video. A demo video for further demonstrating the effectiveness of the developed posture correction system.

## Abbreviations

IP: Internet Protocol
LED: Light-Emitting Diode
MOST: Ministry of Science and Technology
MPU: Microprocessor Unit
